# Smell and Taste in Inflammatory Bowel Disease

**DOI:** 10.1371/journal.pone.0073454

**Published:** 2013-09-25

**Authors:** Silke Steinbach, Wolfgang Reindl, Astrid Dempfle, Anna Schuster, Petra Wolf, Walter Hundt, Wolfgang Huber

**Affiliations:** 1 Department of Otorhinolaryngology, Philipps-University, Marburg, Germany; 2 Medizinische Klinik (Department of Gastroenterology), Klinikum rechts der Isar, Technische Universitaet Muenchen, Munich, Germany; 3 Institut für medizinische Biometrie und Epidemiologie (Institute of Medical Biometry and Epidemiology), Philipps-University, Marburg, Germany; 4 Institut für medizinische Statistik und Epidemiologie (Department of Medical Statistics and Epidemiology), Klinikum rechts der Isar, Technische Universitaet Muenchen, Munich, Germany; 5 Department of Radiology, Philipps-University, Marburg, Germany; Barnard College, Columbia University, United States of America

## Abstract

**Objective:**

To investigate the olfactory/gustatory functions of patients with inflammatory bowel disease (IBD) by smell/taste tests, and to determine if disease activity or medication might influence the olfactory/gustatory functions of patients.

**Patients and Methods:**

In total, 59 IBD patients (37 Crohn's disease (CD) and 22 ulcerative colitis (UC) patients) were studied using “Sniffin' sticks” and “taste strips” for olfactory and gustatory tests, respectively, and compared to healthy controls and published normative data.

**Results:**

Among IBD (CD and UC) patients, the values for odor threshold, but not for odor identification or discrimination, were significantly lower than that of the normative data. Further, these patients showed lower values than the normative taste values and the control group for all tastes, except sour; 57.6% of the IBD patients were hyposmic, while 30.5% were hypogeusic. Subjective self-assessments showed that the patients were not aware of their reduced olfactory/gustatory functions. There were no relevant differences in taste and smell abilities between the CD and UC patients. Disease activity and treatment did not influence the olfactory/gustatory functions.

**Conclusion:**

IBD (CD and UC) patients exhibited significant reductions in the olfactory and gustatory functions. Therefore, patients should be tested by smell/taste tests, in order to be adequately informed of their olfactory/gustatory functions and provided an understanding of how to overcome their limitations, and thus improve their quality of life.

## Introduction

Inflammatory bowel disease (IBD), consisting of the main clinical phenotypes Crohn's disease (CD) and ulcerative colitis (UC), is a chronic relapsing inflammatory disorder of the gastrointestinal tract. CD and UC are complex diseases arising from the interaction of multiple mutations in genes relevant for the regulation of the intestinal immune system, a set of yet poorly defined environmental factors, and the patients' intestinal microbiota [1]. The environmental factors influencing the pathogenesis of IBD are still not fully understood. However, epidemiological studies have shown that the incidence of IBD rises in developing populations adopting industrialized urban lifestyles [2], [3]. The consumption of foods that are low in fiber, high in sugar, and high in animal fat has been proposed to increase the risk for IBD [4], [5]. In particular, CD patients have been shown to consume large amounts of sucrose and refined carbohydrates, whereas they consume few fruits and vegetables [6]–[10]. CD patients commonly have weight loss and nutritional impairment at diagnosis [11]. In CD, 70% of children suffer from weight loss; in UC, 34% of children are affected [12]. In children, IBD, and particularly CD, can lead to an impairment of growth and development. In adults, weight loss is assumed in 65–75% of CD patients and in 18–62% of UC patients [13]. The inflammatory response in IBD patients leads to a reduced resorption of iron; a patient who has a fistula, stenosis, and inflammation can have impaired nutrition leading to significant weight loss, even though the patient might have a relatively large area of uninvolved mucosa in the small intestine. However, some patients continue to experience difficulties in gaining weight, even while they are in complete remission. Although weight loss is a common problem in IBD, some patients gain excessive weight, masking malnutrition problems, such as reduced bone mass or nutritional deficiencies.

Temmel et al. [14] described that patients with reductions in or loss of smell have problems with cooking (73.25%) or appetite loss (56.25%) and often eat rotten (50%) or burnt (30%) food. Thus, such patients often lose weight [15]. However, other patients gain weight [16]. Taste consists of the following qualities: sweet, sour, salty, bitter, and umami. Odors from the food or in the oral cavity reach the olfactory receptor cells in the nose through the retronasal way; all odors are smelled in this manner. It is assumed that 80% of the information in a meal is transmitted through the sense of smell [17]. Therefore, smell seems to be critical to experience a food's flavor. Regarding the higher sucrose consumption in CD patients, the question arises if IBD patients suffer from reductions in the olfactory and gustatory functions and thus consume more sucrose to overcome the deficits and make their meals more interesting. To our knowledge of existing literature the olfactory function (odor identification, discrimination, and threshold) of IBD patients has not been investigated by smell tests so far. Additionally, in this study, the gustatory function of IBD patients was tested by a new taste test employing impregnated taste strips. Former investigations of the gustatory function in CD patients used the Henkin's pipette method [10], [18]–[20]. In this study, the UC patients were also investigated for the olfactory and gustatory functions, and the effects of medication and disease activity on smell and taste were evaluated.

## Patients and Methods

### Patients

In this case-control study, 59 IBD patients (37 CD and 22 UC patients) from a specialized IBD outpatient unit were included. All patients provided written consent for their participation in this study. Ethical approval was obtained for the conduct of the study from the Ethics Committee of the Faculty of Medicine at the Technical University of Munich (Number: 1677/06, amendment 3).

### Controls

Twenty-three healthy volunteers, frequency-matched for age and sex to the patient samples, were used as a control group for the taste evaluation. They underwent exactly the same taste testing procedure as the patients (see below). Twenty-two healthy female volunteers (mean age, 43.6±5.9 years; range, 36–55 years) were tested by the same “Sniffin' Sticks” test battery that was used in the IBD patients to validate whether the smell test results for the IBD patients were comparable with those of the more than 3,000 healthy individuals investigated by Hummel et al. [21]. As there were no significant and relevant differences between the values of the healthy female volunteers (mean values: odor identification, 14.1±1.4; odor discrimination, 13.1±1.4; odor threshold, 9.5±1.9) and the values of the age-matched female group investigated by Hummel et al. [21], the values of IBD patients could be compared with that of the normative data from Hummel et al. [21].

### Subjective assessment

After ear, nose, and throat examinations, the patients assessed subjective topics, such as appetite or the ability to perceive smell and taste. The assessments were based on a visual analogue scale (VAS), with the score for each variable ranging from 0 to 100 (0 =  complete loss of appetite and perception of smell or taste; 100 =  excellent appetite and perception of smell or taste). Thereafter, smell and taste testing was performed.

**Smell testing** was performed using “Sniffin' Sticks”. This test battery consists of 3 subtests for odor identification (ID), odor discrimination (DIS), and odor threshold (THR) and is well validated and highly recommended by the “Working Group Olfaction and Gustation” of the German Society for Otorhinolaryngology, Head and Neck Surgery [21]. Odors were presented in felt-tip pens, the cap was removed, and the tip was positioned approximately 2 cm in front of patient's nostrils for 3 s.

For the ID test, patients separately sniffed 16 pens of different odors. Patients were asked to select the source substance that matched the presented odor from 4 different items in a forced-choice procedure (4-alternative-forced choice). The DIS test kit contained 48 pens arranged in 16 triplets. In each triplet, 2 of the pens contained the same odor, while the third pen contained a different odor. Patients were asked to identify the pen with the different odor (3-alternative-forced choice). The THR kit also contained 48 pens arranged in 16 triplets. In each triplet, 2 of the pens contained a solvent, but the third pen was impregnated with different concentrations of an *n*-butanol solution with a concentration range starting from 4%. The triplets were presented to the patients from the lowest to highest *n*-butanol concentration. From each set, the patients were prompted to choose the pen that differed in smell from the others. They did not need to experience an actual odor, only a slight subtle effect such as a difference in intensity. After choosing the pen containing *n*-butanol from the triplet, the same 3 pens were shuffled and presented again in a random order. If the patient correctly chose the *n*-butanol pen in a triplet the second time, a reversal of the staircase was initiated, until the patient could no longer identify the *n*-butanol pen in the triplet. The THR value is represented as the mean of the last 4 out of the 7 staircase reversal test values. Patients were blindfolded during the THR and DIS tests to avoid visual identification of the pens. *Normative values*: Hummel et al. [21] published normative values for the “Sniffin' Sticks” after investigating more than 3,000 healthy individuals. Both sexes, as well as 4 defined age groups, were considered separately. Additionally, hyposmia was defined as a 10^th^ percentile value or less in healthy individuals aged 16–35 years. Thus, THR values of 6.5 or less in women and of 6.0 or less in men were suggestive of hyposmia (versus normosmia).

**Taste testing** was performed using “taste strips”. A total of 16 filter paper strips were impregnated at 1 end with 1 of the 4 types of taste (sweet, sour, salty, and bitter) in 1 of the following 4 concentrations: 0.05, 0.1, 0.2, or 0.4 g/mL of sucrose (sweet); 0.05, 0.09, 0.165, or 0.3 g/mL of citric acid (sour); 0.016, 0.04, 0.1, or 0.25 g/mL of sodium chloride (salty); and 0.0004, 0.0009, 0.0024, or 0.006 g/mL of quinine hydrochloride (bitter) [22]. Taste strips were placed on 2 sides of the patient's tongue, approximately 1.5 cm from the tip. Patients were instructed to keep their tongues protruded during testing. They were asked to identify the taste stimuli by forced-choice procedures. Correct identification of each taste was scored on a scale of 0–4, with a total of 16 points for 1 side of the tongue and 32 points for both sides of the tongue. *Normative values*: Mueller et al. [22] published normative values for the “taste strips” after investigating 69 healthy individuals. Landis et al. [23] defined hypogeusia as a 10^th^ percentile value or less in healthy individuals aged 18–40 years using a bilateral taste strip test. A value less than 19 in women and less than 17 in men (range, 0–32) would be regarded as hypogeusic (versus normogeusic).

**Statistical analysis** was performed using SPSS software (version 16.0, SPSS Inc., Chicago, IL, USA) and R, version 2.14.1. Descriptive data are presented as absolute and relative frequencies for categorical data and as mean ± standard deviation or median (range) for continuous data. The relationship between the smell and taste test results and the subjective assessment of smell and taste (VAS) was performed using the Spearman's rho correlation coefficient. To compare the patients' smell values with the normative smell data [21], we performed a three-way ANOVA with the factors group (patient or control), sex, and age group to account for the specific age and sex-distributions of our sample. For the comparison of taste values with the normative data [22], the mean of the left and right sides of the tongue was used for the patients, as only unilateral data were available for the published control sample. Total taste values (sum of the 4 taste qualities) of the patients were compared with the normative data using a two-sample t-test, as the normative taste values were not available by sex or age group. Following a significant test of total taste values, post-hoc two-sample t-tests were performed for each individual taste quality to compare the patients' data with the normative data. For comparison of the taste values with our own healthy control group or within subgroups of IBD patients, taste values for the left and right sides of the tongue were added for each individual. Taste values of all 4 taste qualities simultaneously (sweet, sour, salty, and bitter) were compared between the patients and our own healthy control group by multivariate ANOVA (MANOVA), again with the factors group (patient or control) and the sex and covariate age (continuous), using the Pillai-Bartlett statistic. Following a significant MANOVA, univariate post hoc linear models with the same variables were used. For subgroup comparisons (CD versus UC; subgroups based on disease activity, clinical variables, or treatment), analogous MANOVAs were performed for the taste and smell tests. All reported p-values were nominal and should be compared to an adequate significance level to account for multiple testing. For the 2 pre-specified hypotheses that the olfactory and gustatory functions are reduced in patients with IBD, an adequate significance level would be an α = 0.025 each (comparison of smell and taste in the patients versus the control groups). Within the IBD group, subgroup comparisons were performed, covering a total of 28 exploratory hypotheses regarding the influence of 14 independent variables (disease activity, clinical variables or treatment) on 2 dependent variables each (taste and smell). Consequently, these should all be evaluated against a significance level of α = 0.0018.

## Results

### Patient sample

The patients' characteristics, duration and severity of disease, and treatments are summarized in Table 1. No patient had hypo- or hyperactivity of the thyroid gland, diabetes, neurological disorders, tumors or nasal operations in the medical history. Two CD patients and 1 UC patient used angiotensin-converting-enzyme-inhibitor for high blood pressure. None of the patients took medications for depression.

**Table 1:** Patient sample.

	CD patients n = 37	UC patients n = 22
**Sex (n)**		
female	22	10
male	15	12
**Age (years)**		
Mean age ± SD	42.1±13.4	41.5±14.1
Range	25–69	21–65
**BMI**	23.0±3.4	24.8±6.2
**Smokers (n)**	12	2
**Cigarettes (per day)**	3.4±6.2	0.6±1.7
**Alcohol intake, occasional (n)**	3	1
**Duration of disease (years)**	15.4±9.1	15.6±11.9
**Time since last flare (months)**	9.8±7.4	9.4±6.8
**Fistula (n)**	16	0
**Stenosis (n)**	13	2
**Operation of bowel (n)**	21	1
**Extraintestinal manifestation (n)**	10	5
Arthritis	9	5
Uveitis	1	0
Erythema nodosum	0	1
Pyoderma gangraenosum	1	0
**Hemoglobin**	13.3±1.8	12.4±3.2
**Leukocyte count**	8.1±3.1	7.5±3.3
**CRP <0.5 (n)**	18	7
**CDAI (n)**		
active	23	
inactive	14	
**Rachmilewitz (n)**		
active		11
inactive		11
**Truelove Witts (n)**		
moderate		7
severe		8
very severe		7
**Treatment (n)**		
Cortisone	26	13
5-ASA	14	16
Azathioprine	12	6

### Smell and taste test results based on subjective self-evaluation

Table 2 summarizes the subjective assessment results of smell, taste, appetite, parosmia, and phantosmia on VAS. There was no significant correlation between the smell and taste test values and the VAS-assessed results in the IBD patients.

**Table 2:** Subjective assessment of smell and taste on a visual analog scale directly assessed by the patients themselves.

Assessment factor	CD patients	UC patients
**Olfactory function** *	79.7±26.2	80.3±18.0
**Gustatory function** *	80.4±24.5	79.3±18.5
**Appetite** **	69.0±29.1	82.9±19.9
**Smelling of odors not present (phantosmia)** **	1.8±8.6	10.3±18.5
**Things that smell differently (parosmia)** **	6.0±18.4	4.7±14.9
**Food is sweetened more** **	24.9±36.6	7.5±15.7
**Mores salt is used** **	24.6±31.9	11.4±16.4
**Preference for fatty meals** **	14.0±21.6	20.9±22.8
**Preference for more bitter meals** **	8.4±16.0	12.1±19.3

Values represent the mean ± SD on a visual analog scale assessed by the patients themselves.

* score of 0 =  anosmia/ageusia; score of 100 =  hyperosmia/hypergeusia.

** score of 0 =  nothing; score of 100 =  very good/much.

### Smell test results compared to the normative data

IBD patients had significantly lower THR values than those of the healthy controls [21] (p = 4 * 10^−12^, ANOVA adjusted for age group and sex). This effect was also significant if the UC (p = 9 * 10^−5^) and CD (p = 5 * 10^−9^) subgroups were compared separately to the published normative data (Table 3). The values of ID and DIS were not significantly different from that of the normative data (p = 0.56 for ID and p = 0.42 for DIS, adjusted for age group and sex).

**Table 3:** Normative data for the “Sniffin' sticks” [21] and the patients' data, by sex and age group.

	Normative data* female		Normative data* male		IBD female			IBD male			CD female			CD male			UC female			UC male		
	mean	sd	mean	sd	n	mean	sd	n	mean	sd	n	mean	sd	n	mean	sd	n	mean	sd	n	mean	sd
***ID***																						
*16–35 yr*	13.7	1.6	13.5	1.7	14	13.7	1.6	7	12.6	0.8	9	13.9	1.5	6	12.7	0.8	5	13.4	2.1	1	12	
*36–55 yr*	13.5	1.6	13.1	1.9	15	13.6	1.7	14	13.3	3.2	8	13.5	1.7	7	12.1	4.2	5	13.8	1.8	7	14.4	1
*56–75 yr*	12.1	2.3	12.2	2.6	5	12	1.9	4	14	0.9	5	12	1.9	2	13.5	0.7	0			4	14.2	1
***DIS***																						
*16–35 yr*	12.9	1.9	12.6	2.0	14	13.1	1.4	7	10.6	3.9	9	13.2	1.5	6	11.2	3.9	5	12.8	1.3	1	7	
*36–55 yr*	12.5	2.0	11.9	2.2	15	12.4	2.5	14	11.4	3.6	8	12.4	3	7	10.7	4.7	5	12.4	1.7	7	12	2.2
*56–75 yr*	10.7	2.5	10.7	2.8	5	12.4	1.1	4	10.7	1.9	5	12.4	1.1	2	10	1.4	0			4	11	2.2
***THR***																						
*16–35 yr*	9.4	2.6	9.2	3.0	14	6.6	2.3	7	6.3	1.5	9	6.8	2.5	6	6.8	0.9	5	6	2.1	1	3.5	
*36–55 yr*	9.1	3.1	8.4	3.5	15	7.1	2.9	14	4.5	2.5	8	6.7	3.1	7	3.5	2.7	5	7.7	2.8	7	5.5	2
*56–75 yr*	7.4	3.5	7.2	3.6	5	5.2	1.7	4	5.6	3.1	5	5.2	1.7	2	4.9	2.3	0			4	6	3.6

* Hummel et al. [21].

### Taste test results compared to the normative data

The values of total taste were significantly lower in IBD patients than that of the normative data, as reported by Mueller et al. [22] (p = 0.002, t-test). If the UC and CD subgroups were compared separately to the published normative data, the effects were of a similar size but were only marginally significant because of the smaller sample size (p = 0.046 for UC; p = 0.01 for CD). Sweet, sour, salty, and bitter tastes differed slightly between the IBD patients and the published normative data (p = 0.10, p = 0.017, p = 0.016, and p = 0.008 respectively, Table 4). However, the sample by Mueller et al. [22] involved younger participants than the IDB patients in this study (Table 5), and taste ability has been shown to decrease with age [23], thus the observed lower gustatory function could be due to the fact that the patients were older than the controls. Therefore, 23 healthy controls (frequency-matched for sex and age) were tested using taste strips (Table 5). Their taste values were compared with those of the IBD patients, who exhibited significantly lower total taste values (p = 0.001, linear model adjusted for age and sex) and significantly lower values for the individual taste qualities (p = 1.7*10^−5^, MANOVA, and p = 0.002 (sweet), p = 0.0004 (salty), p = 0.0002 (bitter) for post-hoc linear models adjusted for age and sex), except for sour (p = 0.15) (Table 6). The UC and CD patients also separately showed reduced gustatory function in comparison to the healthy controls (p = 0.008 for UC; p = 4.8*10^−6^ for CD, MANOVA).

**Table 4:** Normative data for the “taste strips” [22], data from age- and sex-matched healthy controls and IBD patients (unilateral taste values for normative data, mean of left and right sides of the tongue for the control group and the patients).

	Normative data* n = 69		Own control group		IBD patients n = 59	
			n = 23			
	mean	sd	mean	sd	mean	sd
**Total taste**	12.4	2.3	12.9	1.8	10.6	3.7
**Sweet**	3.3	0.8	3.7	0.7	3	1
**Sour**	3	0.8	2.2	1.1	2.6	1.1
**Salty**	3.1	0.9	3.6	0.6	2.6	1.3
**Bitter**	3	1.1	3.4	0.6	2.4	1.3

* Mueller et al. [22].

**Table 5:** Distribution of the sex and age of the participants investigated by Mueller et[22], and the control group and the patients in this study.

	Control group of Mueller et al. [22] n = 69	Own control group n = 23	IBD patients n = 59	CD patients n = 37	UC patients n = 22
**sex**					
female	36 (52%)	12 (52%)	32 (54%)	22 (59%)	10 (45%)
male	33 (48%)	11 (48%)	27 (46%)	15 (41%)	12 (55%)
**age**					
mean	29	41	42	42	42
minimum	15	20	21	25	21
maximum	75	66	69	69	65
15–34 years	57 (83%)	7 (30%)	19 (32%)	13 (35%)	6 (27%)
35–54 years	10 (14%)	12 (52%)	29 (49%)	17 (46%)	12 (55%)
55–75 years	2 (3%)	4 (18%)	11 (19%)	7 (19%)	4 (18%)

**Table 6:** Taste values (sum of left and right sides of the tongue) in the control group and the patients, by sex and age-group.

	Own control group				IBD patients				CD patients				UC patients			
	female		male		female		male		female		male		female		male	
	mean	sd	mean	sd	mean	sd	mean	sd	mean	sd	mean	sd	mean	sd	mean	sd
**Total taste**																
*16–35 yr*	28.7	2.1	27.2	3.6	26.6	4.8	22.9	5	27.3	3.5	22.8	5.5	25.2	6.9	23	
*36–55 yr*	26.7	3.5	24.8	1	22.9	7.4	18.6	7.7	21.8	8.6	17.7	7.3	24.8	5.4	19.6	8.5
*56–75 yr*	25	1.4	18.5	0.7	16.2	5.2	13.8	7	16.2	5.2	15.5	6.4			13	8.1
**Sweet**																
*16–35 yr*	8	0	7.6	0.9	7.1	1	6.9	0.9	7.1	0.8	6.8	1	7	1.4	7	
*36–55 yr*	7.4	1	7.5	1	6.3	1.9	5.1	2.2	5.9	2.2	4.9	2.5	7	1	5.4	2.1
*56–75 yr*	8	0	4.5	3.5	6	2	4.7	2.3	6	2	6.5	0.7			3.8	2.4
**Sour**																
*16–35 yr*	6.3	0.6	5.2	2.6	6.5	1.7	5.3	1.9	6.7	1.4	5.7	1.8	6.2	2.2	3	
*36–55 yr*	4.4	2.1	4.8	1.9	5.5	1.8	4.6	2.3	5.5	1.8	4.4	2.8	5.6	2.1	4.7	2
*56–75 yr*	2	0	2	2.8	4.8	2.7	2.7	2.3	4.8	2.7	2.5	3.5			2.8	2.2
**Salty**																
*16–35 yr*	7.7	0.6	7.2	1.1	6.9	1.9	5.7	2.3	7.6	0.9	5.5	2.4	5.6	2.6	7	
*36–55 yr*	7.4	1	6.5	1.9	5.2	2.4	4.9	2.7	5	2.6	4.4	2.2	5.6	2.3	5.3	3.1
*56–75 yr*	7	1.4	7	1.4	3.2	3.5	3.3	1.9	3.2	3.5	3.5	2.1			3.2	2.1
**Bitter**																
*16–35 yr*	6.7	1.2	7.2	1.1	6.1	2	5	2.1	6	2.2	4.8	2.2	6.4	1.8	6	
*36–55 yr*	7.4	1	6	0	5.8	2.6	4.1	2.6	5.4	2.7	4	1.4	6.6	2.6	4.1	3.6
*56–75 yr*	8	0	5	1.4	2.2	2.3	3.2	3	2.2	2.3	3	4.2			3.2	3

### Hyposmia and hypogeusia

Of the 59 IBD patients, 34 (57.6%) were hyposmic (THR value below the 10th percentile [21]), 13 of the UC patients (59.1%) and 21 of the CD patients (56.8%). Similarly, 18 of the 59 IBD patients (30.5%) were hypogeusic (total taste score below the 10th percentile [23]), 7 of the UC patients (31.8%) and 11 of the CD patients (29.7%).

### Smell and taste test results in relation to diagnosis, disease activity, and treatment

The UC and CD patients did not differ significantly in their smell test results (p = 0.17, MANOVA, adjusted for age and sex) and their taste test results (p = 0.56, MANOVA, adjusted for age and sex). Age (p = 0.52) and sex (p = 0.04) had no great influence on the smell test results in this sample (MANOVA for age and sex). As expected, taste ability decreased significantly with age (p = 1.4*10^−5^, MANOVA), while there was no relevant effect related to sex (p = 0.08) in our combined case-control sample. Disease activity, as measured using the Truelove-Witts index, the Rachmilewitz index, or the Crohn's disease activity index (CDAI), did not influence the smell and taste test values (all p>0.17, MANOVA models adjusted for age and sex). Further, the values of C-reactive protein, leukocyte count, duration of disease, fistula, stenosis, or operations had no significant influence on the olfactory and gustatory functions in the patients. An extraintestinal manifestation of disease did not influence olfactory function (p = 0.9), but was associated with a slightly differing gustatory function (p = 0.009, MANOVA, adjusted for age and sex), in particular, an improved ability to taste sour (p = 0.0008, post-hoc linear model) but not the other taste qualities (all p >0.14). The use of low-dose cortisone, azathioprine, or 5-aminosalicylic acid (5-ASA) did not influence the olfactory and gustatory functions of the patients (all p >0.12).

## Discussion

This is the first study to investigate the olfactory and gustatory functions in IBD (CD and UC) patients by using validated smell and taste tests. It could clearly be shown that the values of THR, but not ID or DIS, were lower in IBD (CD and UC) patients than those of the healthy individuals. Assuming that the values of THR reflect the peripheral olfactory functions in patients to higher degrees than that of the values of ID or DIS [24], [25], the patients exhibited reduced olfactory functions. By definition, 57.6% of the IBD (CD and UC) patients were hyposmic. Regarding taste, the IBD (CD and UC) patients had lower total taste test values than the participants investigated by Mueller et al. [22] and the healthy control group (matched for age and sex). This reduction in gustatory function was particularly strong for salty and bitter tastes and lower for sweet and sour tastes. By definition, 30.5% of the patients were hypogeusic. In routine clinical practice, taste strips have the major advantages of a long shelf-life, convenience of administration, a short time needed for testing, and possible whole mouth or lateralized taste testing. However, Mueller et al. [22] noticed that the sour taste quality in its lowest concentration was correctly identified by 36% of participants, whereas sweet, salty, and bitter taste qualities were correctly identified by 54%, 51%, and 52%, respectively. This might explain the difficulty that our control group had in correctly identifying the sour taste quality in comparison with the other taste qualities. In addition, Mueller et al. [22] described that some individuals confuse salty and sour. Individual taste blindness must be considered in interpreting taste test values. Although the patients showed reduced functions in the smell and taste tests, they subjectively rated their olfactory and gustatory functions as not being limited on a visual analog scale (VAS). The CD patients rated their olfactory and gustatory functions on the VAS (0 =  no perception of smell and taste; 100  =  excellent perception of smell and taste) as 79.7±26.2 and 80.4±24.5, respectively. Additionally, the UC patients rated their olfactory and gustatory functions on the VAS as 80.3±18.0 and 79.3±18.5, respectively. Using the same method, Fasunla et al. [26] assessed olfactory and gustatory functions on the VAS in 16 healthy individuals and reported values of 83.8±10.4 for smell and of 83.5±10.4 for taste. In addition, Steinbach et al. [27] reported values of 85.9±10.6 for smell and of 82.9±19.2 for taste in 87 patients with gynecological tumors after the operations and before the initiation of chemotherapy. However, patients suffering from Wegener's granulomatosis [26] or hereditary hemorrhagic telangiectasia [28] reported values of 65.7±36.8 or 65.3±27.7, respectively, for smell and 67.2±30.0 or 68.1±25.1, respectively, for taste. This means that IBD patients are hardly aware of their objectively reduced olfactory and gustatory functions. Therefore, it is necessary to test the olfactory and gustatory functions of IBD patients by smell and taste tests, to increase the patients' awareness of their olfactory and gustatory functions, and to discuss the possibilities of overcoming the deficits to improve their quality of life.

On VAS, the CD patients subjectively assessed their appetites as reduced and reported to use more sweeteners and salt for their foods, whereas the UC patients preferred more fatty meals. A low intake of fiber and a high intake of unsaturated fat, as well as high intakes of mono- and disaccharides, have been proposed to have an increased risk for CD and UC [4], [5], [29]. These results confirm the role of diet in the pathogenesis of CD and UC, yet the pathomechanism is still unclear. The interaction of the intestinal microbiota with the intestinal immune system is an essential step in the pathogenesis of CD and UC [1], and new data show the strong influences of diet on the gut microbiota [30]. However, patients suffering from hyposmia and hypogeusia may have higher consumptions of sugar, salt, and fat to make eating more pleasurable. Therefore, some patients with distorted olfactory and gustatory functions gain weight [16], while others complain about having low appetite and weight loss [15]. Distorted olfactory and gustatory functions affect a patients' lives substantially (e.g., malnutrition, reduced detection of environmental hazards, social isolation, and depression) [14]; however, the distorted olfactory and gustatory functions are often not outwardly present.

Among the various dietary interventions for IBD patients, none has shown a striking efficacy, with the possible exception of complete enteral nutrition. Enteral nutrition appears effective in both active and quiescent CD [31], yet it seems to be more effective in pediatric patients [32]. Enteral nutrition may possibly interrupt oral malnutrition in IBD patients, thereby improving the quality of life for these patients. However, for those patients suffering from active CD, corticosteroids have been shown to be superior to enteral nutrition [31], [33]. Although enteral nutrition can be helpful for CD patients, it is limited by inadequate patient compliance and the high cost of enteral formula [31]. It would be worthwhile to investigate in further studies if IBD patients with reduced olfactory and gustatory functions have improvements in their quality of life and in nutritional behavior when adding small amounts of herbs and spices (trigeminal input), glutamate, or other flavors to their foods [34]–[37]. Perhaps taste could be improved by prescribing oral zinc [38], [39].

CD patients can suffer from oral lesions and symptoms (e.g., halitosis, reflux, dry mouth, buccal mucosal swelling or cobblestoning, mucogingivitis, deep linear ulceration, and mucosal tags) [40], [41], whereas nasal involvement is extremely rare and reported only in a few cases of CD [42], [43]. However, in our study, a large proportion of the CD and UC patients were not only hypogeusic, but also hyposmic. It appears that the inflammatory response may possibly cause the distortion of olfactory and gustatory functions. Extraintestinal manifestations show that IBD can affect tissues far away from the site of active intestinal inflammation. Circulating proinflammatory cytokines and activated immune cells trigger these symptoms [44], [45]. Decreased olfactory function is associated with autoimmune disorders such as systemic lupus erythematosus and Sjögren's syndrome [46]. TNF-a has been shown to lead to a reduced olfactory regeneration [47]. Increased levels of circulating TNF-a [48] are present in IBD patients' serum and saliva [49]; therefore, a comparable pathomechanism can be assumed for the effects seen in this study. Recent genetic data [50] suggest a substantial overlap of genetic susceptibility in IBD, as well as other autoimmune diseases such as psoriasis and ankylosing spondylitis. Olfactory receptor gene clusters are located in proximity to key loci for susceptibility to autoimmune diseases, such as the major histocompatibility complex. As with a chromosomal linkage, a functional association may exist. In our study, duration of disease, severity of disease, fistula, stenosis, or operations did not significantly influence the olfactory and gustatory functions in the patients. This is consistent with a study by Zopf et al. 2009 [20], that showed an increased taste threshold for the detection of all 4 taste qualities in 31 active and 27 inactive CD patients, while assessing taste by a 3-drop method with exceeding dilution tests. Decreased taste perception in CD patients was also independent of disease severity in terms of inflammation [20]. However, odorants and taste molecules seem to activate enterochromaffin cells in the bowel tract. Braun et al. [51] found an expression of 4 olfactory receptors in micro-dissected, human, mucosal, enterochromaffin cells, whereas Kidd et al. [52] identified transcripts for bitter and olfactory receptors, as well as transporters for glutamine, glucose, and bile salts in enterochromaffin cells. In the study by Braun et al. [51], an activation of the olfactory receptors in the enterochromaffin cells caused an elevation of the intracellular Ca^2+^ level and a serotonin release. Serotonin release can influence gut motility and secretion. Assuming that the olfactory receptors are equal in the nose and the bowel, decreased THR values in the smell and taste tests may possibly suggest disturbed bowel functions.

It is reported that patients with rheumatoid arthritis (RA) were often hyposmic and hypogeusic [53]. As 9 CD patients and 5 UC patients suffered from arthritis as sources of extraintestinal manifestations (Table 1), we investigated whether patients with and without extraintestinal manifestations showed differences in olfactory and gustatory functions. Only sour taste ability was better in those with an extraintestinal manifestation. The effects of treatment with low-dose oral cortisone, azathioprine, or 5-ASA on olfactory and gustatory functions were minimal and non-significant in our patient sample. A previously published study investigating RA patients showed lower values of THR in patients taking low-dose oral cortisone [53]. However, high doses of oral cortisone are known to be helpful in patients suffering from olfactory dysfunctions [54]. Additionally, reversible dysgeusia has been reported as a negative side effect of azathioprine [55].

It is still not possible to objectively measure parosmia and phantosmia. In our study, patients were asked to subjectively assess parosmia and phantosmia on a VAS (0 = no parosmia/phantosmia; 100 = strong parosmia/phantosmia). The CD and UC patients assessed parosmia as low, whereas the UC patients (10.3±18.5) assumed phantosmia much more intensive than the CD patients (1.8±8.6) or the patients suffering from irritable bowel syndrome (6.4±18.5) and Wegener's granulomatosis (0.5±2.0), using the same method based on a VAS [26], [56]. Reden et al. [57] did, however, show that phantosmia has no prognostic significance in patients suffering from olfactory dysfunctions. In addition, Landis et al. [58] concluded that idiopathic phantosmia does not seem to be a reliable predictor of severe disease.

## Conclusions

IBD (CD and UC) patients exhibited a significant reduction in the olfactory and gustatory functions and a large proportion were hyposmic, hypogeusic, or both. Although the patients had reduced values in the smell and taste tests, they rated their chemosensory functions as not limited on a VAS. Therefore, smell and taste tests should be recommended for IBD patients to measure such deficits, to inform them of the results, and to give them advice on how to overcome limitations and improve their quality of life.
